# Long-term mortality in patients with pulmonary embolism: results in a single-center registry

**DOI:** 10.1016/j.rpth.2023.100280

**Published:** 2023-06-14

**Authors:** Johannes Eckelt, Lukas Hobohm, Marie C. Merten, Charlotta F. Pagel, Ann-Sophie Eggers, Markus H. Lerchbaumer, Karl Stangl, Gerd Hasenfuß, Stavros Konstantinides, Irene Schmidtmann, Mareike Lankeit, Matthias Ebner

**Affiliations:** 1 Clinic of Cardiology and Pneumology, University Medical Center Göttingen, Göttingen, Germany; 2 Center for Thrombosis and Hemostasis (CTH), University Medical Center Mainz, Mainz, Germany; 3 Department of Cardiology, Cardiology I, University Medical Center Mainz, Mainz, Germany; 4 Deutsches Herzzentrum der Charité, Department of Cardiology, Angiology and Intensive Care Medicine, Berlin, Germany; 5 Charité – Universitätsmedizin Berlin, Corporate member of Freie Universität Berlin and Humboldt Universität zu Berlin, Berlin, Germany; 6 Department of Radiology, Campus Charité Mitte (CCM), Charité – Universitätsmedizin Berlin, Berlin, Germany; 7 German Center for Cardiovascular Research (DZHK), Partner site Göttingen, Göttingen, Germany; 8 Department of Cardiology, Democritus University of Thrace, Alexandroupolis, Greece; 9 Institute of Medical Biostatistics, Epidemiology and Informatics (IMBEI), University Medical Center Mainz, Mainz, Germany; 10 German Center for Cardiovascular Research (DZHK), Partner site Berlin, Berlin, Germany

**Keywords:** cancer, mortality, pulmonary embolism, risk factors, venous thromboembolism

## Abstract

**Background:**

While numerous studies have investigated short-term outcomes after pulmonary embolism (PE), long-term mortality remains insufficiently studied.

**Objectives:**

To investigate long-term outcomes in an unselected cohort of patients with PE.

**Methods:**

A total of 896 consecutive patients with PE enrolled in a single-center registry between May 2005 and December 2017 were followed up for up to 14 years. The observed mortality rate was compared with the expected rate in the general population.

**Results:**

The total follow-up time was 3908 patient-years (median, 3.1 years). The 1- and 5-year mortality rates were 19.7% (95% CI, 17.2%-22.4%) and 37.1% (95% CI, 33.6%-40.5%), respectively. The most frequent causes of death were cancer (28.5%), PE (19.4%), infections (13.9%), and cardiovascular events (11.6%). Late mortality (after >30 days) was more frequent than expected in the general population, a finding that was consistent in patients without cancer (the 5-year standardized mortality ratios were 2.77 [95% CI, 2.41-3.16] and 1.80 [95% CI, 1.50-2.14], respectively). Active cancer was the strongest risk factor for death between 30 days and 3 years (hazard ratio [HR], 6.51; 95% CI, 4.67-9.08) but was not associated with later mortality. Death after >3 years was predicted by age (HR, 1.86; 95% CI, 1.51-2.29 per decade), chronic heart failure (HR, 1.66; 95% CI, 1.02-2.70), and anemia (HR, 1.62; 95% CI, 1.09-2.41).

**Conclusion:**

The risk of mortality in patients with PE remained elevated compared with that in the general population throughout the follow-up period. The main driver of long-term mortality during the first 3 years was cancer. After that, mortality was predicted by age, chronic heart failure, and anemia.

## Essentials

• The effects of pulmonary embolism on long-term mortality are insufficiently studied.
• We investigated long-term outcomes in 896 patients with pulmonary embolism followed up for up to 14 years.
• Mortality was higher than expected in the general population throughout the follow-up period.
• This finding was consistent in patients with and without cancer.

## 1 Introduction

Pulmonary embolism (PE) is associated with high morbidity and mortality, making it a major contributor to global disease burden [1,2]. The incidence rates of PE are increasing internationally [1], an effect that has been attributed to the increasing proportion of older individuals in the general population [2] and improved detection due to more frequent use and higher sensitivity of computed tomographic pulmonary angiography [3].

A large body of research has investigated risk factors and therapeutic interventions associated with short-term mortality in patients with acute PE, and the results of these investigations have been transformed into detailed clinical practice guidelines [2,4,5]. It is likely due to these efforts that longitudinal studies of patients with acute PE demonstrated improved short-term mortality over time (assessed at hospital discharge or after 30 days) [6,7].

The high risk of mortality in patients with PE is not limited to the early phase. Long-term mortality rates of >30% after 5 years have been reported, corresponding to a 2.5-fold increase in the risk of mortality compared with that in age- and sex-matched individuals in the general population [8]. However, current knowledge of long-term prognosis of patients with acute PE is still limited. Several studies that investigated long-term outcomes focused on the effects of long-term anticoagulation or thrombolysis and, hence, included a selected study population rather than a cohort of “real-world patients” [[9], [10], [11], [12], [13], [14]]. Other study cohorts were limited to survivors of the acute phase [10,15,16], patients with cancer [17], or elderly individuals aged ≥65 years [18,19]. Most reports that compared mortality rates with those in the general population were based on retrospective analyses of data from health care registries [20,21] or did not account for the high prevalence of cancer in patients with venous thromboembolism (VTE) [8,12].

Hence, the present study aimed to investigate long-term outcomes in an unselected “real-world” cohort of consecutive patients with acute PE, evaluate changes in risk factors and causes of death over the duration of the follow-up period, and provide a comparison with the general population.

## 2 Methods

### 2.1 Study design and definition of outcomes

The Pulmonary Embolism Registry of Göttingen (PERGO) prospectively includes consecutive patients with objectively confirmed acute PE aged ≥18 years admitted to the University Medical Center Göttingen, Germany. The study protocol has been described in detail previously [22,23]. Briefly, patient recruitment is performed by daily screening of new admissions to the emergency department and reports of computed tomography pulmonary angiographies performed. After obtaining informed consent for participation in PERGO, complete data on comorbidities, previous medication, symptoms, results of diagnostic tests, treatment, and clinical course are recorded using a standardized case report form. The modes of data collection did not change over the years. The present analysis included patients enrolled in PERGO between May 2005 and December 2017. We excluded patients with another acute cardiorespiratory illness, such as acute myocardial infarction, left-heart decompensation, or respiratory decompensation responsible for clinical presentation and symptoms as well as recurrent PE (only the first event was included in the analysis).

The study outcome was all-cause death during long-term follow-up. Early mortality was defined as death within 30 days after the diagnosis of PE, whereas late mortality was defined as death >30 days after PE.

Data on patient characteristics, comorbidities, and survival status at the time of hospital discharge were obtained from individually reviewed hospital patient records. The status of these variables (including age and cancer) was determined at the time of hospital discharge; thus, a time-varying analysis was not performed. In patients discharged from hospital alive, long-term survival status was assessed by periodically contacting the responsible community registration offices (in Germany, every town and county has 1 or several registration offices, where every resident has to be registered). Time at risk was calculated from the time of PE diagnosis until death occurred or the last date for which survival status was known. The observation period ended in November 2019.

Participants who were no longer registered (eg, due to emigration) were considered lost to follow-up. In such cases, data were censored on the last date for which survival data were available.

For comparisons of the observed mortality rate in the cohort with the expected mortality rate in the general population, we obtained mortality data for the German reference population from the Human Mortality Database [24].

If death occurred, supplemental information on the cause of death was obtained by reviewing medical records and autopsy reports (if performed) and interviewing the patients’ primary care physicians or family members. The causes of death were independently adjudicated by 2 authors (J.E. and M.E.), and disagreement was resolved by a third author (M.L.).

Diagnostic and therapeutic management was in accordance with the 2008 (September 2008-August 2014) and 2014 (September 2014-April 2018) European Society of Cardiology guidelines [25,26] and local standard operating procedures. The study was conducted in accordance with the amended Declaration of Helsinki and approved by the local independent Ethics Committee of the University Medical Center Göttingen, Germany (protocol number 14/6/10). All patients gave informed written consent for participation in the study.

Renal insufficiency was defined as a glomerular filtration rate of <60 mL/min/1.73 m^2^ of body surface area. Active cancer was defined as a known malignant disease, treatment with antitumor therapy within the last 6 months, metastatic state, or hematologic cancer not in complete remission [27]. Hemodynamic instability and the risk of PE recurrence were assessed according to the criteria proposed by the 2019 European Society of Cardiology guidelines [2].

### 2.2 Statistical analysis

Categorical variables are presented as total numbers and percentages, and continuous variables are presented as medians with IQRs. Associations between binary and categorical variables were analyzed using the Fisher exact test, chi-square test, or Cochran–Armitage test of trend, as appropriate. For analysis of temporal trends in mortality, the enrollment period was *a priori* divided into 4 segments (May 2005-December 2008, January 2009-December 2011, January 2012-December 2014, and January 2015-December 2017). To investigate temporal changes in the importance of risk factors for mortality and causes of death over the duration of follow-up, the follow-up period was also *a priori* divided into 4 segments (0-30 days, 31-365 days, 1-3 years, and >3 years). The Kaplan–Meier analysis was used to evaluate the probability of survival; the log-rank test was used for comparison of subgroups.

The prognostic relevance of baseline characteristics with regard to the study outcome was assessed using univariable Cox proportional hazards models and results expressed as hazard ratios with corresponding 95% CIs. The predictors of mortality identified in the univariable analyses were entered in multivariable Cox proportional hazards models, including all univariable predictors.

Cumulative mortality was obtained using SAS PROC LIFETEST using the Nelson Aalen estimator. CIs are based on log–log transformation. We also computed the expected survival of patients with PE using the Ederer II method [28]. Standardized mortality ratios (SMRs) were calculated to compare the observed all-cause mortality rate in the study cohort with the expected all-cause mortality rate in the general population taking into account sex, age at the time of PE diagnosis, and year of birth. SMRs were calculated for all survivors of the acute phase and the subgroup of survivors of the acute phase without cancer.

A 2-sided significance level of α = .05 was chosen. As this was explorative testing, no adjustments for multiple testing were performed. *P* values were provided for descriptive reasons only and should be interpreted with caution and in connection with effect estimates. Statistical analysis was performed using Statistics Package for Social Sciences (IBM SPSS Statistics, version 27, IBM Corp) and SAS 9.4 using Dickman routine SURVIVAL-PERIOD and included macros [29].

## 3 Results

Between May 2005 and December 2017, 952 patients were enrolled in PERGO. Exclusion criteria applied to 37 (3.9%) patients with significant concomitant acute cardiorespiratory illness and 19 (2.0%) patients with PE recurrence who had already been enrolled in PERGO during an earlier episode of PE. Hence, 896 (94.1%) patients were included in the present analysis.

The total follow-up duration was 3908 patient-years. The median follow-up time per patient was 3.1 (IQR, 1.2-7.1) years, with a maximum duration of 14.0 years. Information on baseline characteristics in the overall cohort is provided in Table 1. Patients with first-time VTE comprised 74.6% of the study cohort. A subgroup analysis of these patients is presented in the Supplementary Material.

**Table 1:** Observed mortality rates in patients stratified according to baseline characteristics and comorbidities.

Characteristic		Whole cohort (n = 896)	Mortality during first 30 d (95% CI)	Mortality during the first year (95% CI)	Mortality during the first 3 y (95% CI)	Mortality during the first 5 y (95% CI)
All patients		8.7% (7.0%-10.7%)	19.7% (17.2%-22.4%)	30.1% (27.0%-33.2%)	37.1% (33.6%-40.5%)	
Sex	Female	52.0%	9.0% (6.7%-11.9%)	19.7% (16.2%-23.5%)	30.2% (25.9%-34.5%)	38.0% (33.2%-42.9%)
	Male	48.0%	8.4% (6.0%-11.3%)	19.7% (16.1%-23.6%)	30.0% (25.6%-34.6%)	36.0% (31.0%-40.9%)
Age (y)	<75	65.7%	6.5% (4.7%-8.7%)	16.0% (13.1%-19.1%)	24.1% (20.6%-27.8%)	28.7% (24.9%-32.7%)
	≥75	34.3%	13.1% (9.6%-17.1%)	26.9% (22.0%-31.9%)	41.8% (35.9%-47.6%)	54.0% (47.4%-60.2%)

Comorbidities						
Obesity (BMI >30 kg/m^2^)	No	65.1% (*n* = 852)	7.2% (5.3%-9.5%)	19.7% (16.5%-23.0%)	31.4% (27.5%-35.3%)	38.5% (34.2%-42.8%)
	Yes	30.0% (*n* = 852)	7.9% (5.0%-11.5%)	15.5% (11.4%-20.1%)	23.7% (18.6%-29.2%)	30.4% (24.5%-36.5%)
Chronic heart failure	No	84.9%	8.1% (6.3%-10.1%)	18.1% (15.4%-20.9%)	26.8% (23.6%-30.1%)	32.9% (29.3%-36.6%)
	Yes	15.1%	12.6% (7.7%-18.8%)	29.0% (21.6%-36.9%)	47.8% (38.8%-56.2%)	58.9% (49.4%-67.1%)
Coronary artery disease	No	82.5%	8.4% (6.6%-10.6%)	18.7% (16.0%-21.6%)	27.9% (24.6%-31.3%)	34.0% (30.3%-37.8%)
	Yes	17.5%	10.2% (6.1%-15.6%)	24.5% (18.0%-31.5%)	40.3% (32.1%-48.2%)	51.1% (42.1%-59.4%)
Prior stroke	No	90.5%	8.1% (6.3%-10.1%)	18.6% (15.9%-21.3%)	28.7% (25.5%-32.0%)	35.7% (32.1%-39.3%)
	Yes	9.5%	15.4% (8.6%-23.9%)	30.9% (21.3%-40.9%)	42.9% (31.9%-53.5%)	49.6% (37.6%-60.4%)
Arterial hypertension	No	37.9%	7.4% (4.9%-10.5%)	18.7% (14.7%-23.1%)	27.0% (22.3%-32.0%)	31.3% (26.1%-36.6%)
	Yes	62.1%	9.6% (7.3%-12.2%)	20.3% (17.1%-23.8%)	32.0% (28.0%-36.1%)	40.8% (36.2%-45.3%)
Chronic pulmonary disease	No	83.6%	8.2% (6.4%-10.3%)	17.8% (15.1%-20.7%)	27.6% (24.3%-31.0%)	33.4% (29.7%-37.0%)
	Yes	16.4%	11.6% (7.0%-17.4%)	29.4% (22.2%-36.9%)	42.8% (34.3%-51.0%)	56.3% (46.6%-64.8%)
Active cancer	No	79.8%	7.3% (5.5%-9.4%)	13.0% (10.6%-15.6%)	21.3% (18.3%-24.6%)	28.6% (25.0%-32.2%)
	Yes	20.2%	14.4% (9.7%-19.9%)	45.9% (38.4%-52.9%)	64.9% (57.0%-71.8%)	71.3% (63.1%-78.0%)
Diabetes mellitus	No	83.5%	7.6% (5.9%-9.7%)	18.3% (15.6%-21.2%)	28.1% (24.8%-31.5%)	35.1% (31.3%-38.8%)
	Yes	16.5%	14.4% (9.2%-20.6%)	26.9% (19.9%-34.3%)	40.1% (31.8%-48.2%)	47.2% (38.3%-55.5%)
Renal insufficiency	No	70.4% (*n* = 840)	5.6% (4.0%-7.7%)	15.7% (12.9%-18.8%)	24.0% (20.5%-27.6%)	29.4% (25.5%-33.4%)
	Yes	29.6% (*n* = 840)	11.3% (7.7%-15.6%)	24.8% (19.6%-30.3%)	40.6% (34.2%-46.9%)	50.8% (43.7%-57.4%)
Anemia	No	61.4% (*n* = 893)	5.7% (3.9%-7.8%)	12.1% (9.6%-15.0%)	21.4% (17.9%-25.1%)	28.0% (23.9%-32.3%)
	Yes	38.6% (*n* = 893)	13.6% (10.2%-17.5%)	31.7% (26.9%-36.7%)	43.8% (38.3%-49.1%)	51.2% (45.4%-56.8%)
Prior VTE	No	74.7%	10.2% (8.0%-12.6%)	22.3% (19.2%-25.5%)	32.6% (28.9%-36.3%)	40.1% (36.0%-44.1%)
	Yes	25.3%	4.5% (2.3%-7.7%)	12.2% (8.3%-16.8%)	22.6% (17.2%-28.5%)	28.4% (22.3%-34.9%)

BMI, body mass index; VTE, venous thromboembolism.

**Figure 1:**
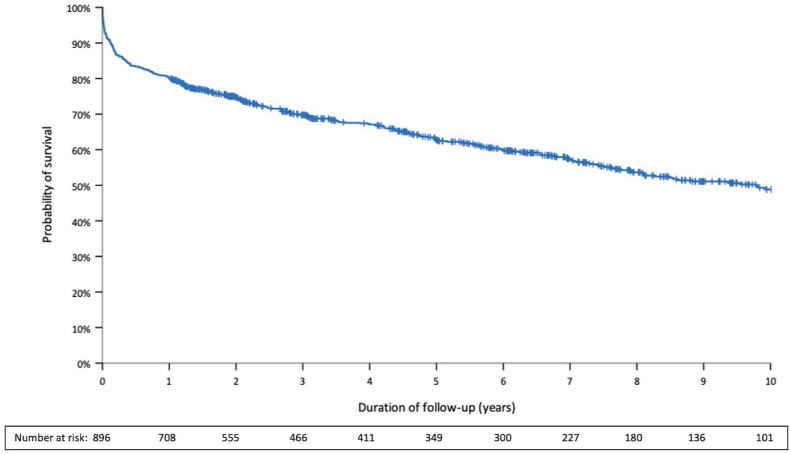
Long-term survival during the first 10 years after acute pulmonary embolism.

Overall, 40.3% of the patients died (92.4/1000 patient-years) during the observation period, with a median time to death of 405 (IQR, 48-1455) days (Figure 1). Within 30 days after PE, 78 patients died, resulting in an early mortality rate of 8.7% (7.0%-10.7%; Table 1). Of those, 50% presented with hemodynamic instability at admission. Late mortality (>30 days after PE) was observed in 283 patients. The 1-, 3-, and 5-year mortality rates were 19.7% (95% CI, 17.2%-22.4%), 30.1% (95% CI, 27.0%-33.2%), and 37.1% (95% CI, 33.6%-40.5%), respectively. No temporal changes in 1-year mortality rates were observed when the study cohort was stratified according to inclusion period (*P* = .47 for this trend, Supplementary Figure S1). A similar finding was obtained in the subgroup of patients with known cancer at the time of PE diagnosis (*P* = .46 for this trend). Patients with PE provoked by a strong transient risk factor (and hence stratified to a low risk of PE recurrence) had a higher probability of long-term survival than patients with an increased risk of recurrence (Supplementary Figure S2).

### 3.1 Causes of mortality

**Figure 2:**
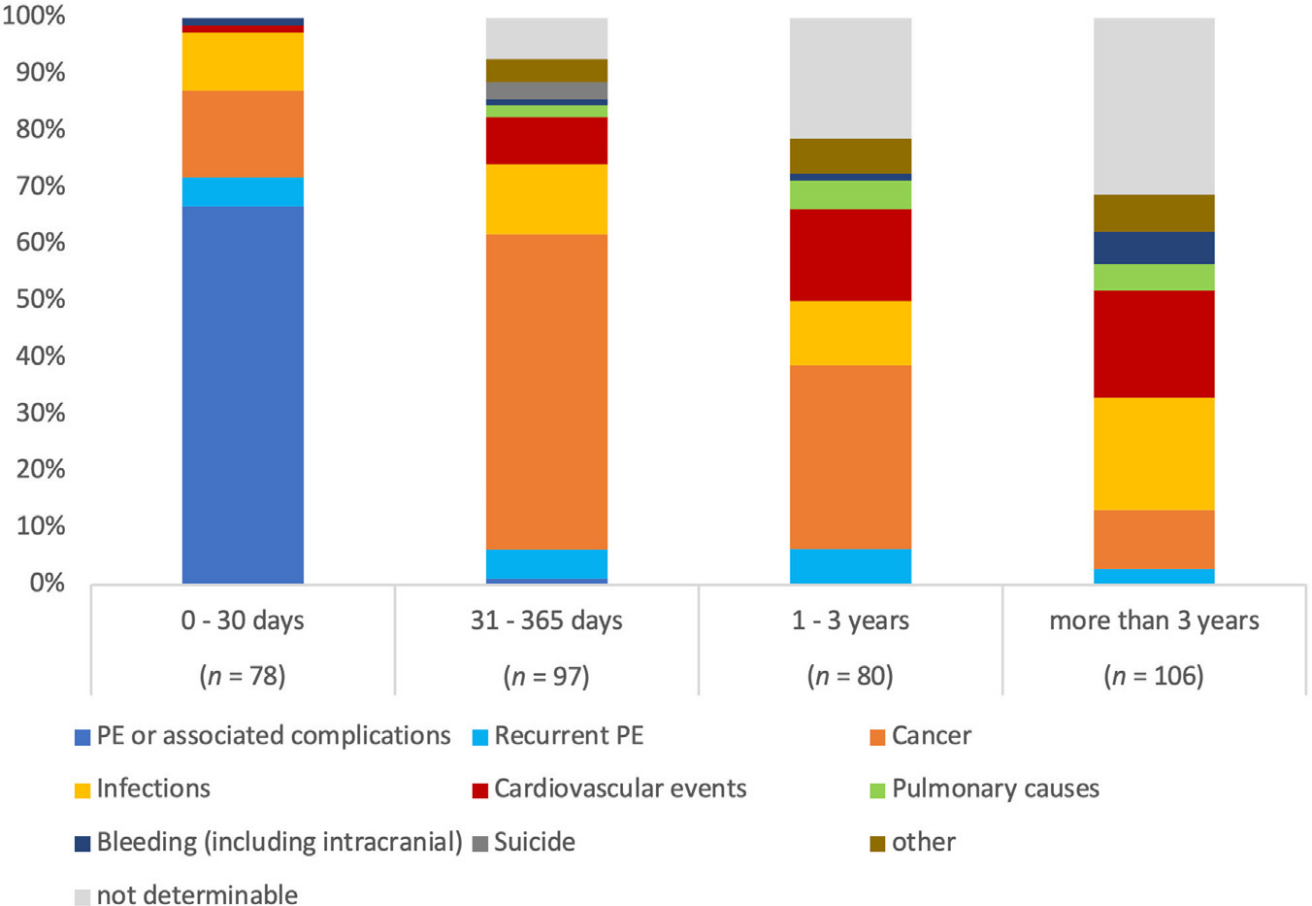
Causes of mortality in patients stratified according to duration of survival. PE, pulmonary embolism.

As presented in Table 2, the most frequent cause of death was cancer (28.5%; 26.4/1000 patient-years), followed by PE (19.4% [14.7% due to initial PE and 4.7% due to recurrent episodes of PE]; 17.9/1000 patient-years), infections (13.9%; 12.8/1000 patient-years), and cardiovascular events (11.6%; 10.7/1000 patient-years). PE was the leading cause of early mortality, accounting for 66.7% of all deaths ≤30 days after PE (Figure 2, left column). Cancer was the most frequent cause of death between 31 days and 3 years after PE, while infections and cardiovascular events were predominant reasons for mortality after >3 years (Figure 2, middle and right columns). Of note, during the first 3 years after PE, more deaths were due to PE recurrence than due to bleeding.

**Table 2:** Causes of mortality during long-term follow-up.

Causes of death	
PE or associated complications	14.7% (*n* = 53)
Recurrent PE	4.7% (*n* = 17)
Cancer	28.5% (*n* = 103)
Infectious causes	13.9% (*n* = 50)
Cardiovascular causes	11.6% (*n* = 42)
Pulmonary causes	3% (*n* = 11)
Bleeding (including intracranial)	2.5% (*n* = 9)
Suicide	0.8% (*n* = 3)
Other	4.4% (*n* = 16)
Not determinable	15.8% (*n* = 57)

PE, pulmonary embolism.

### 3.2 Influence of cancer on mortality

At the time of PE diagnosis, 181 (20.2%) patients had a diagnosis of active tumor disease, of whom 78 (43.1%) had known metastases. A detailed description of the subgroups of patients with PE with and without cancer can be found in the Supplementary Material.

**Figure 3:**
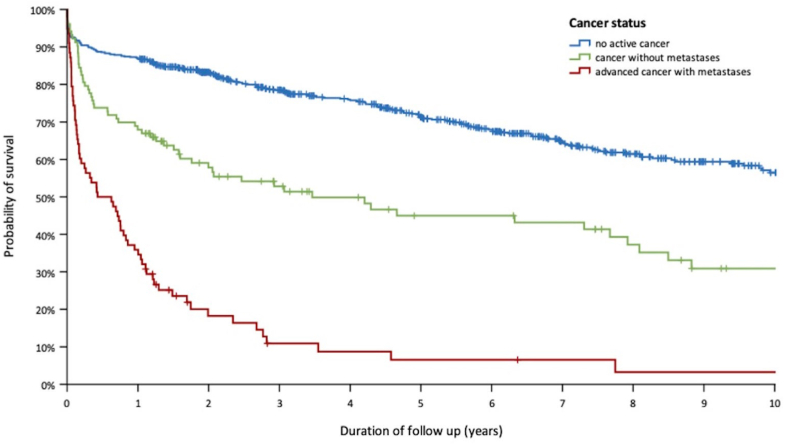
Probability of survival in patients with pulmonary embolism stratified according to cancer status.

As shown in Figure 3 and Supplementary Table S1, a higher overall mortality rate was observed in patients with PE with active cancer than in patients with PE without tumor disease (291.2/1000 patient-years vs 67.0/1000 patient-years, respectively; *P* < .001). As expected, patients with PE with advanced cancer were more likely to die during follow-up than patients without known metastases (*P* < .001; Figure 3).

### 3.3 Predictors of long-term mortality after PE

Univariable and multivariable predictors of overall long-term mortality and late mortality are presented in Table 3. In multivariable analyses, late mortality was predicted by active cancer, chronic pulmonary disease, anemia, chronic heart failure, and age at the time of PE diagnosis, while obesity was associated with a reduced risk of long-term mortality. Similar results were observed in the subgroup of patients without cancer (Table 3, right column). A more detailed analysis of mortality predictors during different time periods after acute PE is provided in Table 4. Active cancer at the time of PE diagnosis presented the strongest risk factor for death during the first 3 years after PE but was not associated with increased risk of mortality during the later follow-up period.

**Table 3:** Predictors of overall and late mortality in the whole cohort and in patients without active cancer.

Characteristic	Overall mortality during follow-up (*n* = 361)		Late mortality after >30 d (*n* = 283)		Late mortality after >30 d in patients without active cancer (*n* = 180)	
	Univariable model HR (95% CI)	Multivariable model HR (95% CI)^a^	Univariable model HR (95% CI)	Multivariable model HR (95% CI)^a^	Univariable model HR (95% CI)	Multivariable model HR (95% CI)^a^
Age per decade	**1.50 (1.38-1.63)**	**1.45 (1.30-1.61)**	**1.56 (1.42-1.72)**	**1.49 (1.32-1.68)**	**2.17 (1.87-2.53)**	**1.97 (1.66-2.33)**
Sex (female)	0.93 (0.75-1.16)	-	0.91 (0.72-1.16)	-	0.88 (0.65-1.19)	-
Obesity (BMI >30 kg/m^2^)	**0.77 (0.60-0.98)**	**0.75 (0.57-0.98)**	**0.69 (0.53-0.91)**	**0.65 (0.48-0.87)**	**0.68 (0.48-0.96)**	**0.66 (0.45-0.98)**

Comorbidities						
Chronic heart failure	**2.13 (1.66-2.74)**	**1.45 (1.09-1.92)**	**2.30 (1.75-3.04)**	**1.67 (1.23-2.28)**	**3.57 (2.60-4.90)**	**1.84 (1.31-2.59)**
Coronary artery disease	**1.74 (1.35-2.23)**	1.14 (0.86-1.50)	**1.83 (1.39-2.42)**	1.12 (0.82-1.52)	**2.54 (1.83-3.52)**	1.04 (0.73-1.48)
Prior stroke	**1.95 (1.44-2.64)**	**1.45 (1.05-1.99)**	**1.85 (1.31-2.62)**	1.34 (0.93-1.92)	**2.38 (1.60-3.53)**	1.23 (0.82-1.86)
Arterial hypertension	**1.48 (1.17-1.87)**	0.83 (0.64-1.08)	**1.54 (1.19-1.99)**	0.93 (0.70-1.24)	**2.25 (1.59-3.19)**	0.90 (0.61-1.32)
Chronic pulmonary disease	**1.66 (1.28-2.17)**	**2.03 (1.54-2.68)**	**1.70 (1.26-2.28)**	**2.16 (1.58-2.93)**	**1.97 (1.39-2.81)**	**2.22 (1.51-3.27)**
Active cancer	**3.61 (2.88-4.53)**	**3.76 (2.95-4.80)**	**4.07 (3.17-5.23)**	**4.22 (3.23-5.51)**	-	-
Diabetes mellitus	**1.51 (1.16-1.96)**	**1.33 (1.01-1.75)**	**1.41 (1.05-1.90)**	1.27 (0.93-1.73)	**1.66 (1.17-2.37)**	1.20 (0.83-1.73)
Renal insufficiency	**1.99 (1.59-2.48)**	1.17 (0.91-1.52)	**1.97 (1.54-2.52)**	1.07 (0.80-1.41)	**3.35 (2.47-4.54)**	1.17 (0.82-1.65)
Anemia	**2.30 (1.85-2.85)**	**1.80 (1.43-2.25)**	**2.27 (1.79-2.89)**	**1.84 (1.44-2.36)**	**1.84 (1.36-2.50)**	**1.62 (1.19-2.21)**
Prior VTE	**0.76 (0.59-0.98)**	**0.71 (0.55-0.92)**	0.84 (0.64-1.11)	-	1.00 (0.72-1.39)	-

Bold text indicates statistically significant results.

BMI, body mass index; HR, hazard ratio; VTE, venous thromboembolism.

a Including all univariable predictors.

**Table 4:** Predictors of late mortality during different time periods after acute pulmonary embolism.

Characteristic	Mortality between 31 and 365 d (*n* = 97)		Mortality between 1 and 3 y (*n* = 80)		Mortality after >3 y (*n* = 106)	
	Univariable model HR (95% CI)	Multivariable model HR (95% CI)^a^	Univariable model HR (95% CI)	Multivariable model HR (95% CI)^a^	Univariable model HR (95% CI)	Multivariable model HR (95% CI)^a^
Age per decade	**1.30 (1.12-1.51)**	**1.30 (1.08-1.55)**	**1.52 (1.27-1.82)**	**1.37 (1.10-1.71)**	**1.94 (1.62-2.32)**	**1.86 (1.51-2.29)**
Sex (female)	1.06 (0.70-1.60)	-	0.95 (0.60-1.48)	-	0.77 (0.52-1.14)	
Obesity (BMI >30 kg/m^2^)	0.61 (0.37-1.00)	-	0.65 (0.38-1.10)	-	0.81 (0.53-1.24)	

Comorbidities						
Chronic heart failure	**1.79 (1.10-2.91)**	1.48 (0.87-2.53)	**2.61 (1.59-4.29)**	**1.98 (1.14-3.43)**	**2.64 (1.68-4.16)**	**1.66 (1.02-2.70)**
Coronary artery disease	1.46 (0.90-2.38)	-	**1.97 (1.19-3.26)**	1.23 (0.71-2.12)	**2.15 (1.36-3.40)**	1.10 (0.67-1.80)
Prior stroke	**1.83 (1.02-3.30)**	**1.99 (1.06-3.71)**	1.51 (0.75-3.02)	-	**2.16 (1.25-3.74)**	1.17 (0.66-2.08)
Arterial hypertension	1.02 (0.67-1.56)	-	1.61 (0.98-2.64)	-	**2.16 (1.41-3.32)**	1.07 (0.68-1.67)
Chronic pulmonary disease	**2.04 (1.28-3.25)**	**2.47 (1.53-3.99)**	**1.76 (1.03-3.02)**	**1.83 (1.06-3.17)**	1.32 (0.76-2.28)	
Active cancer	**7.68 (5.04-11.70)**	**7.84 (4.96-12.42)**	**4.35 (2.72-6.97)**	**5.17 (3.12-8.55)**	**1.74 (1.03-2.94)**	1.38 (0.80-2.35)
Diabetes mellitus	1.37 (0.82-2.29)	-	1.50 (0.86-2.60)	-	1.38 (0.85-2.25)	
Renal insufficiency	1.46 (0.95-2.24)	-	**2.24 (1.43-3.52)**	1.45 (0.86-2.43)	**2.35 (1.57-3.50)**	0.87 (0.55-1.37)
Anemia	**3.48 (2.26-5.36)**	**2.17 (1.38-3.40)**	**1.88 (1.20-2.95)**	1.42 (0.89-2.26)	**1.81 (1.22-2.68)**	**1.62 (1.09-2.41)**
Prior VTE	0.61 (0.36-1.03)	-	0.83 (0.50-1.38)	-	1.08 (0.72-1.62)	

Bold text indicates statistically significant results.

BMI, body mass index; HR, hazard ratio; VTE, venous thromboembolism.

a Including all univariable predictors.

### 3.4 Long-term mortality compared with that in the general population

**Figure 4:**
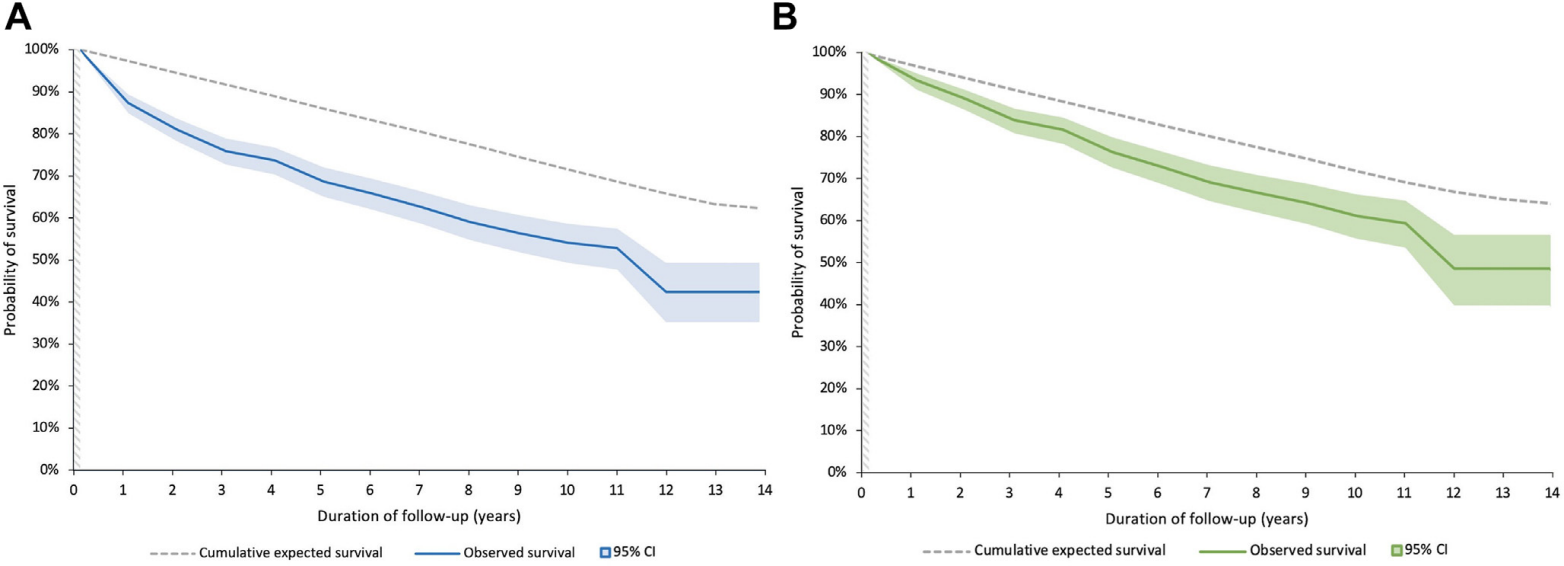
Observed and expected long-term survival in (A) survivors of the acute phase of pulmonary embolism and (B) survivors of the acute phase of pulmonary embolism without cancer.

In survivors of the acute phase (initial 30 days after PE diagnosis), the rates of long-term mortality were higher than the expected mortality rate in the general population taking into account sex, age, and year of birth (Figure 4A). The SMRs after 3 and 5 years were 3.35 (95% CI, 2.88-3.88) and 2.77 (95% CI, 2.41-3.16), respectively. After 5 years, SMRs remained elevated until the end of the follow-up period (1.41; 95% CI, 1.08-1.81). This finding was consistent in the subgroup of patients without cancer at the time of PE diagnosis (Figure 4B), in whom 3- and 5-year SMRs of 1.94 (95% CI, 1.56-2.37) and 1.80 (95% CI, 1.50-2.14) were observed. Further subgroup analyses in patients stratified according to sex and age provided similar results (Table 5).

**Table 5:** Standardized mortality rates in 30-day survivors.^a^

Characteristic	SMR after 3 y (95% CI)	SMR after 5 y (95% CI)
All patients	3.35 (2.88-3.88)	2.77 (2.41-3.16)
Cancer status		
Active cancer	15.82 (12.75-19.42)	11.63 (9.44-14.18)
Non-cancer	1.94 (1.56-2.37)	1.80 (1.50-2.14)
Sex		
Female	3.38 (2.75-4.12)	2.86 (2.38-3.42)
Male	3.33 (2.69-4.09)	2.67 (2.20-3.27)
Age (y)		
<75	7.25 (5.93-8.78)	5.41 (4.50-6.46)
≥75	2.02 (1.61-2.49)	1.77 (1.45-2.14)

SMR, standardized mortality rate.

a For the calculation of SMRs, observed mortality was compared with expected mortality in the general population taking into account sex, age, and year of birth.

## 4 Discussion

Many patients experience the acute phase after PE as a dramatic and potentially life-threatening event. Based on data from a real-world single-center cohort of 896 patients with PE consecutively enrolled over a 12-year period and followed up for up to 14 years, we demonstrated that mortality rates remain high even after the immediate risks of the acute phase have subsided. While most early deaths can be attributed to PE or associated complications, cancer becomes the main cause of death after the acute phase up until 3 years after PE, whereas cardiovascular events and infections gain in importance after that. Importantly, although the most pronounced increase in mortality compared with that in the general population was observed within the first 5 years after PE, SMRs remained elevated until the end of the follow-up period. This finding was consistent in both the overall study population and patients without cancer.

### 4.1 Risk of long-term mortality in patients with PE

The reported long-term mortality rates in patients with PE vary widely between studies, likely due to multifactorial causes such as differences in age and cancer status between study cohorts, observation period, and performance characteristics of national health care systems. While historical data based on patients in the United States who developed VTE between 1966 and 1990 suggest 5-year mortality rates of >60% [20], a study based on patients recruited from outpatient anticoagulation clinics in the Netherlands between 1999 and 2004 observed a 5-year mortality rate of only 10% [16]. One reason for the low mortality observed in this latter study was likely the study design, which excluded patients with in-hospital mortality during their PE-related stay. Furthermore, the authors reported a relatively young cohort (median age, 50 years vs 69 years in our cohort) with low rates of cancer and cardiovascular comorbidities [16]. In contrast, the 5-year mortality rate of 37.1% in our cohort of German patients with PE included between 2005 and 2017 is more in line with findings obtained in patients enrolled in an Australian single-center registry between 2000 and 2007 (31.6%) [8] and PE cases included in a Canadian regional health database between 2004 and 2012 (26.0%) [21].

In our present analysis, 71% of the observed deaths occurred during the first 3 years after PE. Hence, even after exclusion of patients who died during the initial 30 days after PE, we observed a >3-fold increase in mortality in our cohort compared with the expected rate in the general population during that time period. Although attenuated in magnitude, this effect continued throughout the follow-up period. Patients who survived for 5 years after PE diagnosis were still 40% more likely to die during further follow-up than expected based on age, sex, and year of birth. This finding is in contrast to an earlier investigation by Naess et al. [30], who reported that the mortality rate in patients with non-cancer-associated VTE normalized after 3 years.

Not surprisingly and in accordance with previous studies [8,21], late mortality (occurring >30 days after PE) was associated with cancer, chronic cardiopulmonary disease, anemia, and age. There was an inverse correlation between obesity and late mortality, a finding in accordance with the “obesity paradox” described in different cardiovascular diseases, including PE [31,32]. Of note, this finding does not infer a causal relationship but rather reflects differences in baseline risk in those exposed to the respective risk factors. As reported previously [30], we could confirm that PE with a low risk of recurrence (eg, after major surgery or trauma [2]) was associated with significantly higher chances of long-term survival compared with other causes of PE.

In patients with cancer, death was not only twice as frequent as that in patients without malignancy but also occurred sooner after PE. As a result, the observed mortality per 1000 patient-years was 7 times higher than that in patients without cancer (291.2/1000 patient-years vs 37.3/1000 patient-years, respectively). Of note, the importance of active cancer at the time of PE as a risk factor changed significantly over the duration of follow-up. Active cancer was the most important risk factor for late mortality until 3 years after PE. During this time, almost 2 out of 3 patients with cancer died, with exceptionally high mortality rates in patients with known metastases. In patients who survived the first 3 years after PE, known cancer at the time of PE diagnosis was not associated with higher mortality during further follow-up. This finding probably reflects a case of survivor bias: most cancers in these patients were likely either cured or in remission, thus affecting the risk of mortality less than cardiovascular risk factors and age.

### 4.2 Causes of long-term mortality

Not surprisingly, cancer was the main driver of overall long-term mortality in our cohort (28.5%), confirming the findings of earlier reports [16,19]. In contrast to our findings, Ng et al. [8] reported that cardiovascular diseases (including PE) are the most frequent cause of late (postdischarge) mortality in patients with PE, accounting for 36% of deaths. Despite a similar distribution of baseline rates of cardiovascular comorbidities and cancer in their study and our cohort, we observed a substantially lower rate of cardiovascular or PE-related mortality in our cohort (19.5% after >30 days). As discussed earlier, this lower rate in our more recent study was likely due to multifactorial causes but may also have been influenced by the declining cardiovascular death rate of the overall population [33].

Similar to the temporal changes in risk factors described above, the causes of death also shifted in significance depending on the duration of survival after PE. More specifically, while most early deaths could be attributed to PE or associated complications, cancer was the predominant cause of death between 31 days and 3 years after PE, whereas cardiovascular events and infections were the most frequent reasons for mortality after >3 years. Of note, death due to bleeding was rare in our cohort (2.5%) and occurred >3 years after PE in 6 of 9 cases. In contrast, death due to PE recurrence occurred almost twice as often (4.7%). Importantly, during the first year after PE, we observed 9 fatal PE recurrences but only 2 fatal bleedings. Although detailed information on the duration of anticoagulation in individual patients was not available, this may suggest underuse or an insufficient duration of anticoagulation treatment in our cohort.

### 4.3 Strengths and limitations

Our report adds substantially to previous studies. First, we reported a large cohort of well-characterized patients and provided longer follow-up data (up to 14 years after PE) compared with previous reports [8,19]. Second, we included consecutive patients at the time of PE and, hence, excluded bias that may have influenced the results of studies recruiting only from outpatient clinics [15,16]. However, due to the single-center study design, a certain selection bias could not be excluded, limiting the generalizability of the study findings. Surveillance bias may have impacted the number of cancer diagnoses and the reported causes of death. Due to the long follow-up period, the causes of death could not be determined in 15.8% of the patients. This may limit the accuracy of our findings, especially in patients who died >3 years after PE. Data on the race/ethnicity of the study patients were not available, and hence, we could not evaluate differences in long-term mortality between ethnic groups. Due to limitations in the available data in the control cohort of individuals from the general population, we could only match for sex, age, and year of birth but not for the presence of cancer and other comorbidities. This should be taken into account when interpreting the findings of our study. Finally, it is important to emphasize that the observed hazard ratios are not causally interpretable and that survivor bias might explain some of the observed changes in the importance of risk factors over the duration of follow-up.

## 5 Conclusion

Our study showed that the risk of mortality in patients with PE was not limited to the acute phase but remained elevated compared with that in the general population throughout the follow-up period of up to 14 years. Importantly, the risk factors and causes of death shifted in importance depending on the duration of follow-up. Although cancer, at the time of PE diagnosis, was the strongest overall predictor of death during follow-up, its effect on mortality was limited to the first 3 years after PE. After that, cardiovascular causes and infections were the most frequent causes of death, and mortality was predicted by age, chronic heart failure, and anemia at baseline.
