# Supplementary material for: Long-term mortality in patients with pulmonary embolism: results in a single-center registry
Source: Res Pract Thromb Haemost. 2023 Jun 14;7(5):100280. doi: 10.1016/j.rpth.2023.100280 (PMC10439384; doi:10.1016/j.rpth.2023.100280)

**SUPPLEMENTAL MATERIAL**

**Long-term mortality in pulmonary embolism: results in a single-center registry**

**Supplemental Results**

*Patients with cancer-related PE*

Cancer was diagnosed prior to PE in 90.1% of patients (median time between cancer diagnosis and PE 128 [30-539] days), while in the remaining 9.9% the diagnosis was established during the PE-related hospital stay. Most frequent tumor types were colorectal carcinoma (11.0%), lung cancer (9.9%), head and neck cancer (9.9%), breast cancer (9.4%) and brain cancer (8.8%). Of note, in patients with cancer-related death during follow-up, presence of tumor disease was known at the time of PE diagnosis in 88.3%.

We did not observe a difference in median age between patients with and without active cancer at the time of PE (66 [IQR 59-75] vs. 70 [IQR 54-78]), p=0.37). A comparison of other baseline characteristics and outcomes is provided in **Supplemental** **Table S1**. Patients with known cancer less frequently suffered from cardiovascular comorbidities compared to PE patients without cancer. PE was an incidental finding in 10.5% of cancer patients and in 1.3% of patients without cancer (p<0.001).

Cancer was identified as the cause of mortality in 70.5% of patients with active tumor disease at the time of PE diagnosis. In contrast, of patients without known tumor disease at the time of PE diagnosis only 5.2% died due to cancer; cardiovascular events were the most frequent cause of mortality after >30 days in this group (21.7%).

*Subgroup analyses in patients first time venous thromboembolism*

Of 896 patients included in the main analysis, 669 had no prior history of venous thromboembolism (VTE).

The total follow-up duration in patients with first time VTE was 2,722 patient years. The median follow-up time per patient was 2.8 (IQR 1.1-6.8) years, with a maximum duration of 13.4 years. Information on baseline characteristics in patients with first time and with recurrent VTE is provided in **Supplemental** **Table S2.**

Overall, 41.6% of patients with first time VTE died (102.1/1,000 patient years) during the observation period with a median time to death of 275 (IQR 35-1121) days (**Supplemental** **Figure S3**). Within 30 days after PE, 68 patients died, resulting in an early mortality rate of 10.2% (8.0%-12.6%; **Supplemental** **Table S3)**. Of these, 48.5% presented with hemodynamic instability at admission. Late mortality (>30 days after PE) was observed in 210 patients.
One-, three- and five-year mortality rates were 22.3% (19.2%-25.5%), 32.6% (28.9%-36.3%) and 40.1% (36.0% - 44.1%), respectively.

At the time of PE diagnosis, 144 (21.25%) patients had a diagnosis of active tumor disease, of whom 61 (42.4%) had known metastases. A higher overall mortality rate was observed in PE patients without prior VTE with active cancer compared those without tumor disease (331.2/1,000 patient years vs. 71.9/1,000 patient years; p<0.001).

Causes of death in patients with first time venous thromboembolism

The most frequent cause of death was cancer (22.2%; 29.4/1,000 patient years), followed by PE (17.2% [13.0% due to initial PE and 4.2% due to a recurrent episode of PE]; 23.0/1,000 patient years), infections (9.1%; 12.1/1,000 patient years) and cardiovascular events (8.9%; 11.8/1,000 patient years).

PE was the leading cause of early mortality, accounting for 67.6% of all deaths **≤**30 days after PE (**Supplemental Figure 4**, left column). Cancer was the most frequent cause of death between 31 days and three years after PE, while infections and cardiovascular events were the predominant reasons for mortality after more than three years (**Supplemental Figure 4**, middle and right columns).

*Predictors of long-term mortality after PE with first time venous thromboembolism*

Predictors of overall long-term mortality and late mortality are presented in **Supplemental** **Table S4** and a more detailed analysis of mortality predictors during different time periods after acute PE is provided in **Supplemental** **Table S5**.

*Long-term mortality compared to the general population*

In survivors of the acute phase (initial 30 days after PE diagnosis), rates of long-term mortality were higher than expected in the general population taking into account sex, age and year of birth (**Supplemental** **Figure 5A**). SMR after three and five years were 3.83 (95% CI 3.23-4.52) and 3.23 (95% CI 2.78-3.74), respectively. In 5-year survivors, SMR remained numerically elevated until the end of the follow-up period (1.39 [95% CI 0.98-1.98]), although this finding did not reach statistical significance. Consistent results were obtained in the subgroup of patients without cancer at the time of PE diagnosis (**Supplemental** **Figure 5B**), in whom 3- and 5-year SMR of 2.09 (95% CI 1.63-2.64) and 2.00 (95% CI 1.63-2.43) were observed.

**Supplemental Tables**

**Supplemental Table S1:** Comparison of characteristics, comorbidities and outcomes of patients stratified according to cancer status at time of PE diagnosis

|  | **Patients with active cancer (n=181)** | **Patients without cancer (n=715)** | **p-value** |
| --- | --- | --- | --- |
| Age ≥ 75 years | 51 (28.2%) | 256 (35.8%) | 0.05 |
| Sex (female) | 86 (47.5%) | 380 (53.1%) | 0.18 |
| Obesity (BMI >30 kg/m^2^) | 49 (27.5%), n=178 | 220 (32.6%), n=674 | 0.19 |
| Incidental PE | 19 (10.5%) | 9 (1.3%) | **<0.001** |
| **Comorbidities** |  |  |  |
| Chronic heart failure | 14 (7.7%) | 121 (16.9%) | **0.002** |
| Coronary artery disease | 22 (12.2%) | 135 (18.9%) | **0.033** |
| Prior stroke | 12 (6.6%) | 73 (10.2%) | 0.14 |
| Arterial hypertension | 105 (58.0%) | 451 (63.1%) | 0.21 |
| Chronic pulmonary disease | 26 (14.4%) | 121 (16.9%) | 0.41 |
| Diabetes mellitus | 21 (11.6%) | 127 (17.8%) | **0.046** |
| Renal insufficiency | 30 (17.3%), n=173 | 219 (32.8%), n=667 | **<0.001** |
| Anaemia | 111 (62.0%), n=179 | 235 (32.9%), n=714 | **<0.001** |
| Prior VTE | 37 (20.6%) | 190 (26.6%) | 0.09 |
| **PE severity** |  |  |  |
| Hemodynamic instability on admission | 13 (7.2%) | 85 (11.9%) | 0.07 |
| **Outcome** |  |  |  |
| Mortality during follow-up  30-day mortality rate  1-year mortality rate  3-year mortality rate  5-year mortality rate | 14.4% (9.7%-19.9%)  45.9% (38.4%-52.9%)  64.9% (57.0%-71.8%)  71.3% (63.1%-78.0%) | 7.3% (5.5%-9.4%)  13.0% (10.6%-15.6%)  21.3% (18.3%-24.6%)  28.6% (25.0%-32.2%) |  |

Abbreviations: BMI denotes body mass index; VTE, venous thromboembolism; PE, pulmonary embolism.

**Supplemental** **Table S2:** Comparison of characteristics, comorbidities and hemodynamic status at admission in PE patients with first and recurrent VTE

|  | **All patients**  **(n=896)** | **Patients with first time VTE (n=669)** | **Patients with recurrent VTE (n=227)** | **p-value** |
| --- | --- | --- | --- | --- |
| Age ≥ 75 years | 307 (34.3%) | 228 (34.1%) | 79 (34.8%) | 0.84 |
| Sex (female) | 466 (52.0%) | 345 (51.6%) | 121 (53.3%) | 0.65 |
| Obesity (BMI >30 kg/m^2^) | 269 (31.6%), n=852 | 201 (31.9%), n=630 | 68 (30.6%), n=222 | 0.73 |
| **Comorbidities** |  |  |  |  |
| Chronic heart failure | 135 (15.1%) | 101 (15.1%) | 34 (15.0%) | 0.97 |
| Coronary artery disease | 157 (17.5%) | 113 (16.9%) | 44 (19.4%) | 0.39 |
| Prior stroke | 85 (9.5%) | 62 (9.3%) | 23 (10.1%) | 0.7 |
| Arterial hypertension | 556 (62.1%) | 414 (61.9%) | 142 (62.6%) | 0.86 |
| Chronic pulmonary disease | 147 (16.4%) | 109 (16.3%) | 38 (16.7%) | 0.88 |
| Active cancer | 181 (20.2%) | 144 (21.5%) | 37 (16.3%) | 0.09 |
| Diabetes mellitus | 148 (16.5%) | 109 (16.3%) | 39 (17.2%) | 0.76 |
| Renal insufficiency | 249 (29.6%), n=840 | 178 (28.7%), n=620 | 71 (32.3%), n=220 | 0.32 |
| Anemia | 346 (38.6%), n=893 | 275 (41.3%), n=666 | 71 (31.3%) | **0.007** |
| **PE severity** |  |  |  |  |
| Hemodynamic instability on admission | 98 (10.9%) | 85 (12.5%) | 13 (6.1%) | **0.004** |

Abbreviations: PE denotes pulmonary embolism; VTE, venous thromboembolism; BMI, body mass index.

**Supplemental** **Table S3:** Observed mortality rates in PE patients with first time VTE stratified according to baseline characteristics and comorbidities

|  |  |  | **Mortality during  first 30 days (95% CI)** | **Mortality during  first year (95% CI)** | **Mortality during  first three years (95% CI)** | **Mortality during**  **first five years (95% CI)** |
| --- | --- | --- | --- | --- | --- | --- |
| First time VTE patients |  | n=669 | 10.2% (8.0%-12.6%) | 22.3% (19.2%-25.5%) | 32.6% (28.9%-36.3%) | 40.1% (36.0%-44.1%) |
| Gender | female | 51.6% | 11.0% (8.0%-14.6%) | 23.3% (18.9%-27.8%) | 34.1% (29.0%-39.3%) | 41.7% (35.9%-47.4%) |
|  | male | 48.4% | 9.3% (6.5%-12.8%) | 21.2% (16.9%-25.9%) | 31.0% (25.8%-36.3%) | 38.3% (32.4%-44.1%) |
| Age (years) | <75 | 65.9% | 7.3% (5.1%-10.0%) | 18.1% (14.6%-21.8%) | 26.3% (22.2%-30.6%) | 31.3% (26.7%-36.0%) |
|  | ≥ 75 | 34.1% | 15.8% (11.4%-20.8%) | 30.3% (24.4%-36.3%) | 45.1% (38.1%-51.9%) | 58.6% (50.5%-65.8%) |
| **Comorbidities** |  |  |  |  |  |  |
| Obesity | no | 68.1% (n=630) | 8.0% (5.6%-10.8%) | 22.1% (18.2%-26.1%) | 33.1% (28.5%-37.7%) | 40.1% (35.0%-45.1%) |
| (BMI >30 kg/m2) | yes | 31.9% (n=630) | 10.0% (6.3%-14.6%) | 17.4% (12.5%-23.0%) | 27.1% (21.0%-33.7%) | 35.7% (28.4%-43.1%) |
| Chronic heart failure | no | 84.9% | 9.4% (7.1%-11.9%) | 20.9% (17.7%-24.4%) | 29.9% (26.0%-33.8%) | 36.8% (32.4%-41.1%) |
|  | yes | 15.1% | 14.9% (8.7%-22.5%) | 29.7% (21.1%-38.8%) | 46.9% (36.6%-56.5%) | 57.0% (46.0%-66.5%) |
| Coronary artery | no | 83.1% | 9.8% (7.5%-12.4%) | 21.2% (17.9%-24.7%) | 30.9% (26.9%-34.9%) | 37.7% (33.2%-42.1%) |
| disease | yes | 16.9% | 12.4% (7.1%-19.2%) | 27.5% (19.6%-36.0%) | 41.1% (31.5%-50.4%) | 51.4% (40.7%-61.1%) |
| Prior stroke | no | 90.7% | 9.3% (7.1%-11.7%) | 20.9% (17.8%-24.2%) | 31.0% (27.2%-34.8%) | 38.4% (34.1%-42.7%) |
|  | yes | 9.3% | 19.4% (10.6%-30.1%) | 35.5% (23.8%-47.4%) | 48.2% (34.9%-60.4%) | 55.6% (41.0%-68.0%) |
| Arterial hypertension | no | 38.1% | 7.9% (5.0%-11.7%) | 21.0% (16.2%-26.2%) | 28.6% (23.0%-34.4%) | 32.7% (26.6%-38.8%) |
|  | yes | 61.9% | 11.6% (8.7%-14.9%) | 23.0% (19.1%-27.2%) | 35.2% (30.4%-40.0%) | 44.9% (39.4%-50.3%) |
| Chronic pulmonary | no | 83.7% | 9.7% (7.4%-12.3%) | 20.8% (17.6%-24.3%) | 30.5% (26.6%-34.5%) | 36.9% (32.5%-41.3%) |
| disease | yes | 16.3% | 12.9% (7.4%-19.9%) | 29.5% (21.2%-38.3%) | 43.4% (33.5%-52.9%) | 56.8% (45.3%-66.8%) |
| Active cancer | no | 78.5% | 8.8% (6.6%-11.4%) | 15.0% (12.0%-18.2%) | 23.1% (19.5%-27.0%) | 30.8% (26.5%-35.3%) |
|  | yes | 21.5% | 15.3% (10.0%-21.7%) | 48.6% (40.2%-56.5%) | 67.2% (58.3%-74.6%) | 74.1% (64.9%-81.3%) |
| Diabetes mellitus | no | 83.7% | 8.8% (6.6%-11.3%) | 21.1% (17.8%-24.5%) | 30.4% (26.5%-34.4%) | 37.4% (32.9%-41.8%) |
|  | yes | 16.3% | 17.4% (11.0%-25.1%) | 28.4% (20.3%-37.1%) | 43.3% (33.5%-52.8%) | 53.2% (42.5%-62.7%) |
| Renal insufficiency | no | 71.3% (n=620) | 6.8% (4.7%-9.4%) | 18.2% (14.8%-22.0%) | 26.4% (22.3%-30.8%) | 32.4% (27.7%-37.1%) |
|  | yes | 28.7% (n=620) | 12.9% (8.5%-18.4%) | 27.1% (20.7%-33.8%) | 43.7% (35.9%-51.2%) | 54.2% (45.7%-62.0%) |
| Anemia | no | 58.9% (n=666) | 6.9% (4.7%-9.7%) | 14.1% (10.9%-17.8%) | 23.9% (19.5%-28.4%) | 31.2% (26.1%-36.4%) |
|  | yes | 41.1% (n=666) | 14.9% (11.0%-19.4%) | 33.8% (28.3%-39.5%) | 45.0% (38.8%-50.9%) | 52.5% (45.9%-58.7%) |

Abbreviations: PE denotes pulmonary embolism; VTE, venous thromboembolism; BMI, body mass index.

**Supplemental** **Table S4**: Predictors of overall and late mortality in PE patients with first time VTE

|  | **Overall mortality during follow-up  (n=278)** | | **Late mortality after >30 days  (n=210)** | | **Late mortality after >30 days  in patients without active cancer (n=127)** | |
| --- | --- | --- | --- | --- | --- | --- |
|  | **Univariable model HR (95% CI)** | **Multivariable model HR (95% CI)*** | **Univariable model HR (95% CI)** | **Multivariable model  HR (95% CI)*** | **Univariable model HR (95% CI)** | **Multivariable model  HR (95% CI)*** |
| Age per decade | **1.43 (1.31-1.56)** | **1.37 (1.23-1.53)** | **1.48 (1.34-1.63)** | **1.45 (1.28-1.64)** | **1.57 (1.39-1.79)** | **1.56 (1.34-1.8)** |
| Sex (female) | 0.98 (0.77-1.24) | - | 0.91 (0.70-1.20) | - | 0.78 (0.56-1.08) | - |
| Obesity (BMI >30 kg/m^2^) | 0.81 (0.62-1.07) | **-** | **0.71 (0.52-0.98)** | **0.69 (0.49-0.98)** | 0.84 (0.58-1.21) | - |
| **Comorbidities** |  |  |  |  |  |  |
| Chronic heart failure | **1.80 (1.36-2.39)** | 1.21 (0.87-1.68) | **1.86 (1.35-2.57)** | 1.29 (0.89-1.86) | **2.10 (1.43-3.08)** | 1.30 (0.85-1.98) |
| Coronary artery disease | **1.46 (1.09-1.94)** | 1.23 (0.88-1.71) | **1.82 (1.32-2.57)** | 1.29 (0.89-1.87) | **1.64 (1.10-2.45)** | 1.04 (0.68-1.61) |
| Prior stroke | **1.86 (1.32-2.63)** | 1.46 (0.99-2.13) | **1.76 (1.17-2.66)** | 1.31 (0.83-2.05) | 1.54 (0.91-2.59) |  |
| Arterial hypertension | **1.47 (1.14-1.89)** | **0.73 (0.54-0.98)** | **1.46 (1.09-1.95)** | 0.80 (0.56-1.13) | **1.83 (1.28-2.61)** | 0.92 (0.62-1.37) |
| Chronic pulmonary disease | **1.55 (1.16-2.08)** | **2.08 (1.50-2.88)** | **1.64 (1.17-2.29)** | **2.42 (1.66-3.53)** | **1.52 (1.01-2.30)** | **1.56 (1.00-2.44)** |
| Active cancer | **3.49 (2.72-4.45)** | **4.43 (3.34-5.86)** | **4.39 (3.32-5.81)** | **5.37 (3.93-7.34)** | **-** | **-** |
| Diabetes mellitus | **1.51 (1.13-2.02)** | 1.27 (0.93-1.74) | **1.35 (0.96-1.90)** | - | 1.45 (0.96-2.18) | 1.20 (0.83-1.73) |
| Renal insufficiency | **1.88 (1.46-2.42)** | **1.38 (1.02-1.86)** | **1.85 (1.39-2.47)** | 1.26 (0.89-1.79) | **2.00 (1.41-2.82)** | 1.00 (0.68-1.48) |
| Anemia | **2.13 (1.68-2.70)** | **1.77 (1.36-2.30)** | **2.09 (1.59-2.74)** | **1.78 (1.33-2.38)** | **1.80 (1.29-2.51)** | **1.71 (1.21-2.41)** |

* including all univariable predictors

Abbreviations: PE denotes pulmonary embolism; VTE, venous thromboembolism; HR, hazard ratio; CI, confidence interval; BMI, body mass index.

**Supplemental** **Table S5**: Predictors of late mortality in PE patients with first time VTE during different time periods after acute PE

|  | **Mortality between 31 and 365 days (n=80)** | | **Mortality between 1 and 3 years (n=59)** | | **Mortality after more than 3 years (n=71)** | |
| --- | --- | --- | --- | --- | --- | --- |
|  | **Univariate model HR (95% CI)** | **Multivariate model HR (95% CI)*** | **Univariate model HR (95% CI)** | **Multivariate model HR (95% CI)*** | **Univariate model HR (95% CI)** | **Multivariate model HR (95% CI)*** |
| Age per decade | **1.28 (1.1-1.48)** | **1.31 (1.1-1.56)** | **1.48 (1.22-1.79)** | **1.36 (1.08-1.73)** | **1.79 (1.47-2.19)** | **1.73 (1.37-2.17)** |
| Sex (female) | 1.05 (0.68-1.63) | - | 1.13 (0.68-1.88) | **-** | 0.65 (0.41-1.04) |  |
| Obesity (BMI >30 kg/m^2^) | **0.52 (0.3-0.92)** | 0.61 (0.34-1.07) | 0.84 (0.48-1.47) | **-** | 0.84 (0.5-1.41) |  |
| **Comorbidities** |  |  |  |  |  |  |
| Chronic heart failure | 1.41 (0.81-2.48) | **-** | **2.31 (1.3-4.1)** | **1.92 (1.03-3.57)** | **2.06 (1.19-3.56)** | 1.33 (0.75-2.35) |
| Coronary artery disease | 1.4 (0.82-2.39) | **-** | 1.5 (0.81-2.78) | **-** | 1.72 (0.97-3.04) | **-** |
| Prior stroke | 1.64 (0.85-3.18) | **-** | 1.58 (0.72-3.48) | **-** | **2.12 (1.05-4.27)** | 1.22 (0.57-2.6) |
| Arterial hypertension | 0.89 (0.57-1.4) | **-** | 1.7 (0.97-2.99) | **-** | **2.29 (1.37-3.85)** | 1.08 (0.61-1.89) |
| Chronic pulmonary disease | 1.66 (0.98-2.81) | **-** | 1.74 (0.94-3.22) | **-** | 1.51 (0.81-2.8) |  |
| Active cancer | **7.1 (4.54-11.12)** | **6.7 (4.11-10.91)** | **4.77 (2.83-8.06)** | **6.4 (3.68-11.14)** | **1.9 (1.02-3.54)** | 1.75 (0.93-3.28) |
| Diabetes mellitus | 1.01 (0.55-1.86) | **-** | 1.74 (0.95-3.16) | **-** | 1.44 (0.8-2.59) |  |
| Renal insufficiency | 1.34 (0.83-2.16) | **-** | **2.23 (1.32-3.77)** | 1.69 (0.91-3.16) | **2.26 (1.38-3.7)** | 0.97 (0.55-1.72) |
| Anemia | **3.11 (1.96-4.92)** | **2.00 (1.22-3.29)** | 1.61 (0.96-2.7) | **-** | **1.69 (1.06-2.69)** | 1.46 (0.89-2.37) |

* including all univariate predictors

Abbreviations: PE denotes pulmonary embolism; VTE, venous thromboembolism; HR, hazard ratio; CI, confidence interval; BMI, body mass index.

**Supplemental Figures**

**Supplemental** **Figure S1:** Long-term survival during the first 10 years after acute PE in patients stratified according to inclusion period


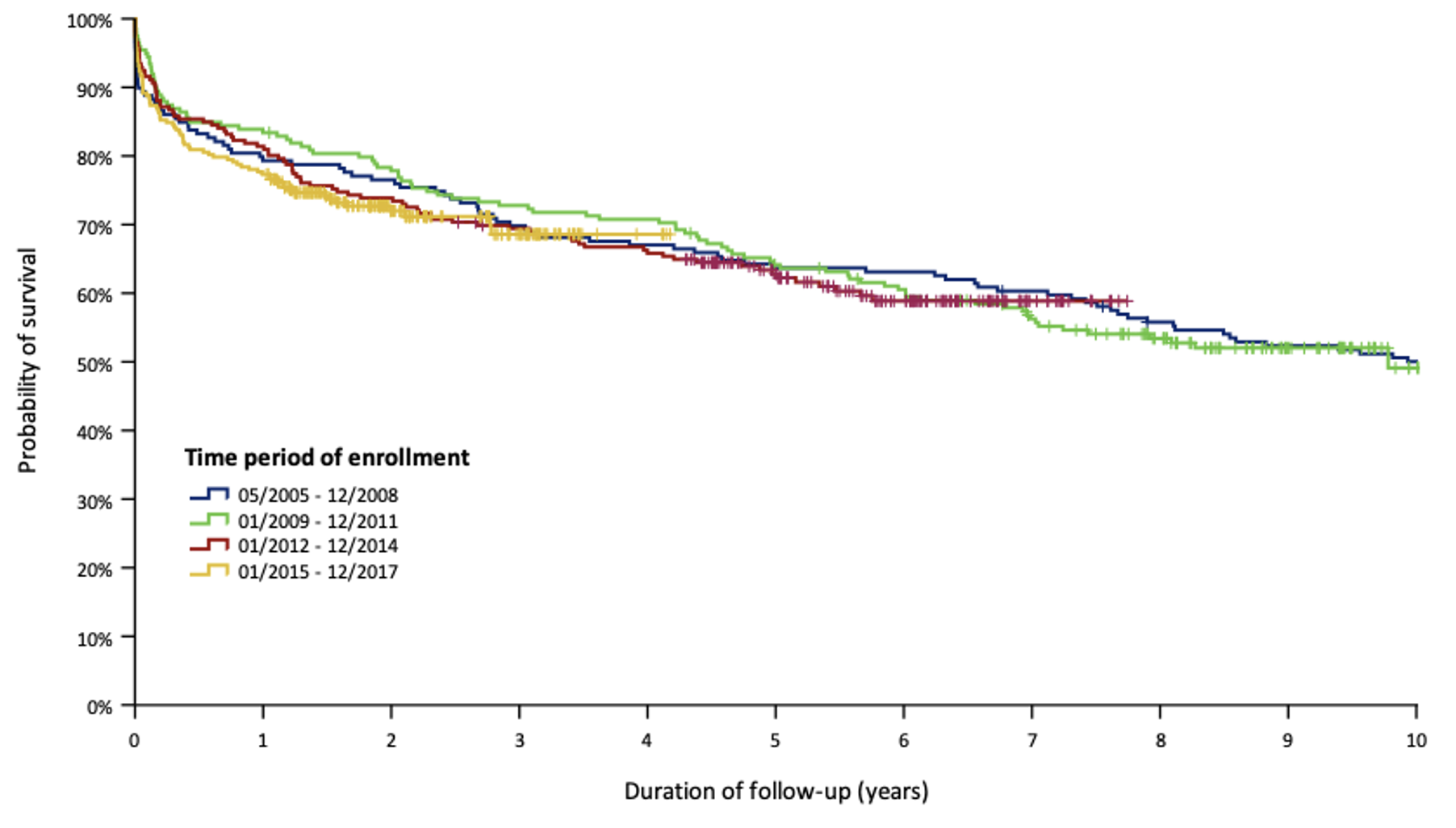


PE denotes pulmonary embolism.

**Supplemental Figure S2:** Long-term survival during the first 10 years after acute PE in acute phase survivors stratified according to risk of PE recurrence


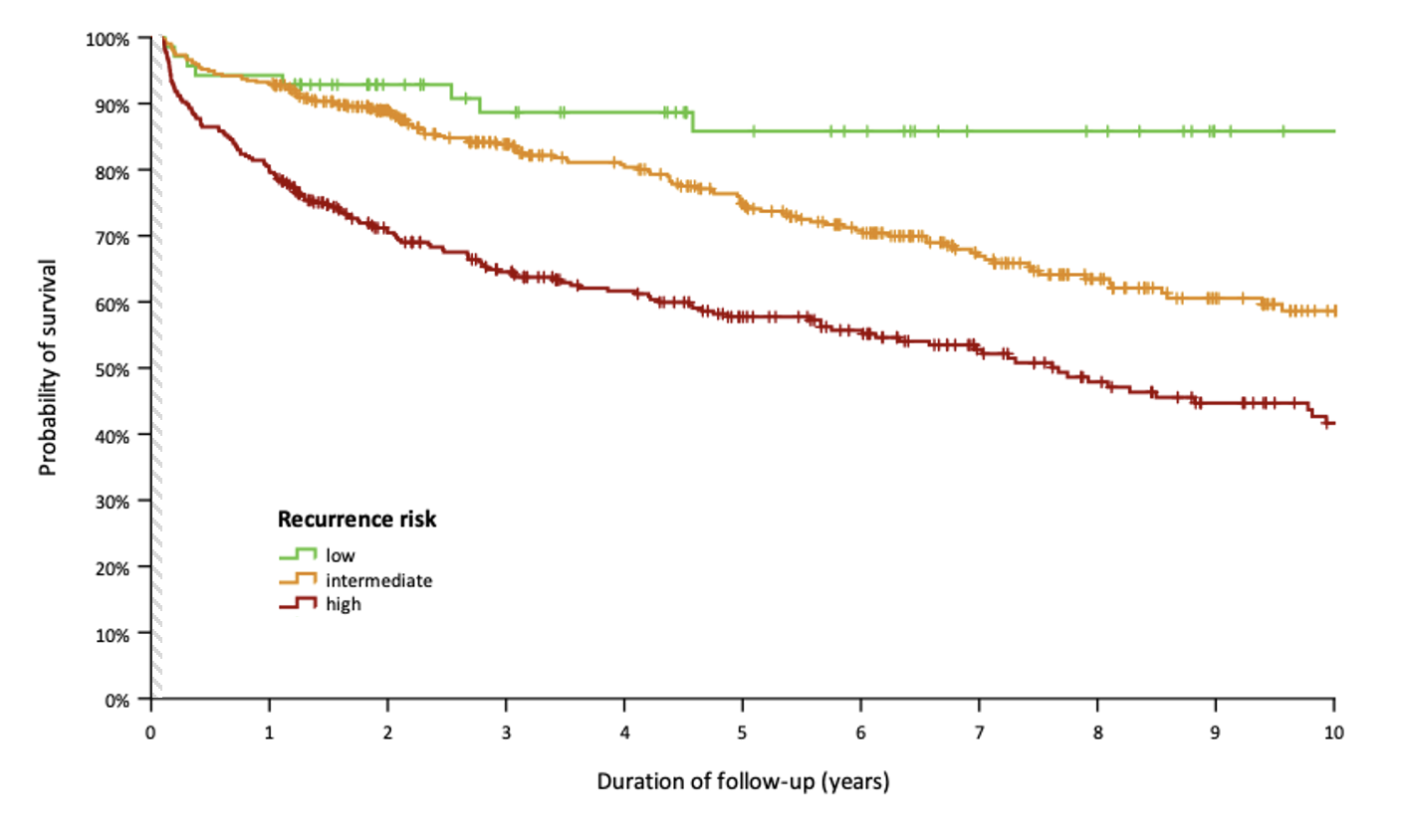


Risk of long-term PE recurrence was calculated according to the classification provided in the 2019 European Society of Cardiology guidelines (Konstantinides et al. Eur Heart J 2019).

PE denotes pulmonary embolism.

**Supplemental Figure S3:** Long-term survival during the first 10 years after acute PE in patients with first time VTE


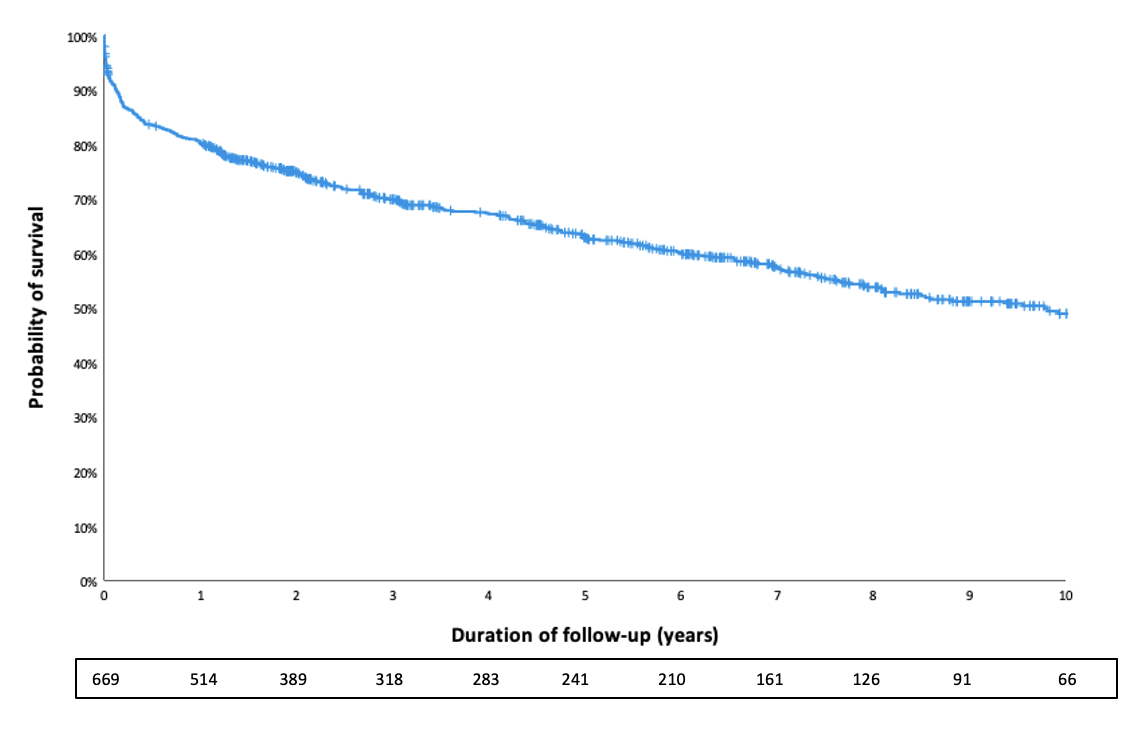


PE denotes pulmonary embolism; VTE, venous thromboembolism.

**Supplemental Figure 4**: Causes of mortality in PE patients with first time VTE patients stratified according to duration of survival

PE denotes pulmonary embolism; VTE, venous thromboembolism.

**Supplemental Figure S5**: Observed and expected long-term survival (A) in first time VTE patients who survived of the acute phase of PE and (B) first time VTE acute phase survivors without cancer

(A)


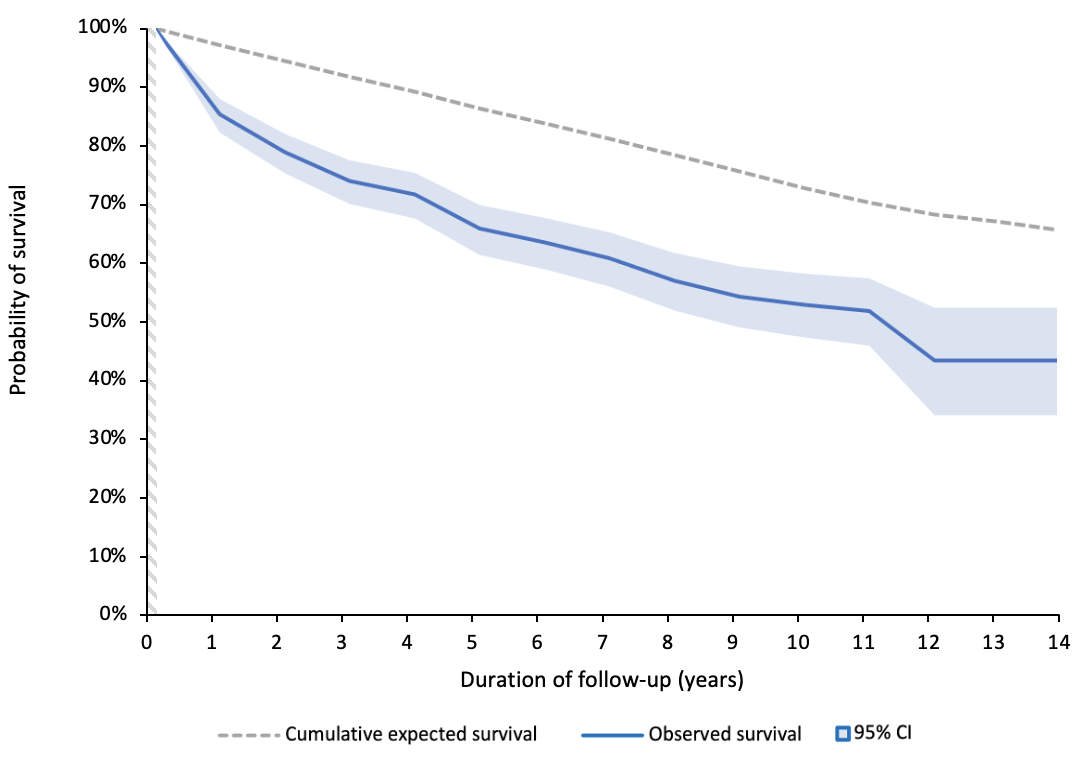


PE denotes pulmonary embolism; VTE, venous thromboembolism.

(B)


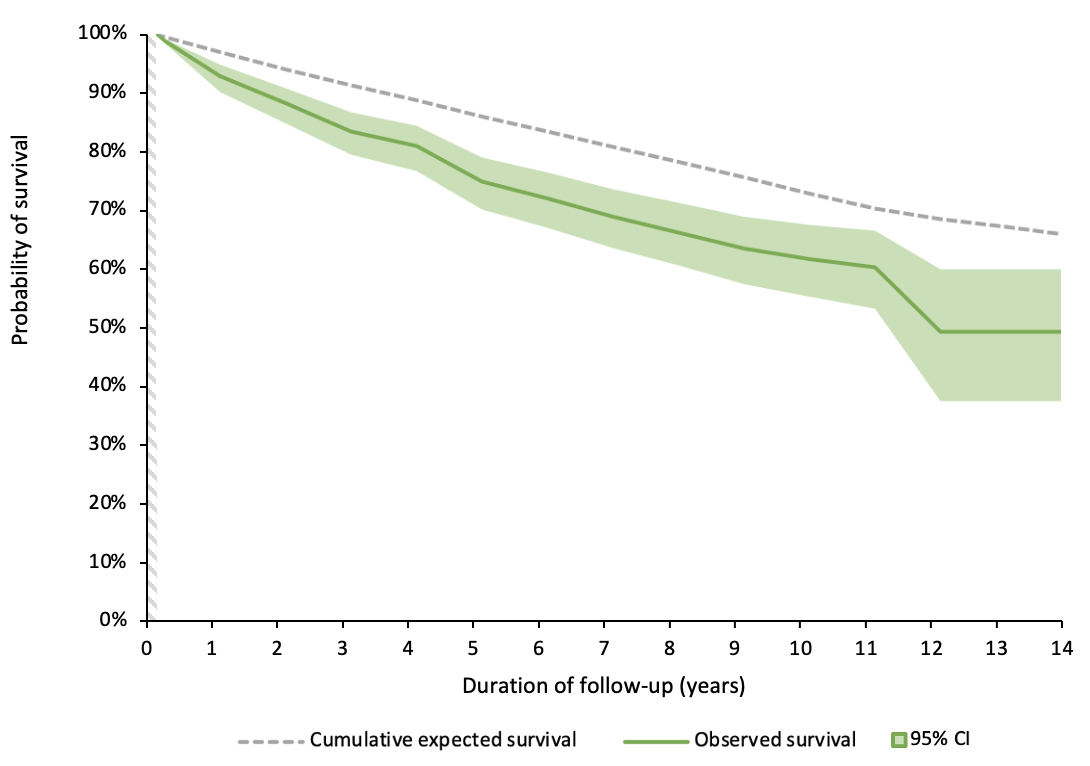

Supplement: Supplementary material [file mmc1.docx]
